# A New Ionone Glycoside and Three New Rhemaneolignans from the Roots of *Rehmannia glutinosa*

**DOI:** 10.3390/molecules200815192

**Published:** 2015-08-20

**Authors:** Meng Li, Xiaolan Wang, Xiaoke Zheng, Jianchao Wang, Wei Zhao, Kai Song, Xuan Zhao, Yanli Zhang, Haixue Kuang, Weisheng Feng

**Affiliations:** 1 School of Pharmacy, Heilongjiang University of Chinese Medicine, Harbin 150040, China; E-Mails: limeng31716@163.com (M.L.); hxkuang@hotmail.com (H.K.); 2 Collaborative Innovation Center for Respiratory Disease Diagnosis and Treatment & Chinese Medicine Development of Henan Province, Zhengzhou 450046, China; E-Mails: wxl_@163.com (X.W.); zhengxk.2006@163.com (X.Z.); 15238397097@163.com (J.W.); 18236159652@163.com (W.Z.); songky5901@163.com (K.S.); lilas616@hotmail.com (X.Z.); zyl2013hnzy@163.com (Y.Z.); 3 School of Pharmacy, Henan University of Traditional Chinese Medicine, Zhengzhou 450046, China

**Keywords:** *Rehmannia glutinosa*, ionone glycoside, rhemaneolignans, the protective effects of cardiomyocytes

## Abstract

A new ionone glycoside, frehmaglutoside I (**1**), and three new rhemaneolignans **A**–**C** (**2**–**4**) were isolated from the 95% EtOH extract of the roots of *Rehmannia glutinosa.* Their structures were determined by extensive spectroscopic (UV, IR, HR-ESI-MS, 1D and 2D NMR) analyses. In addition, these compounds were evaluated for their protective effects on cardiocytes impaired by doxorubicin in H9c2 cells. Among them, compounds **1**–**3** exhibited protective effects against DOX-induced cardiotoxicity.

## 1. Introduction

**Figure 1:**
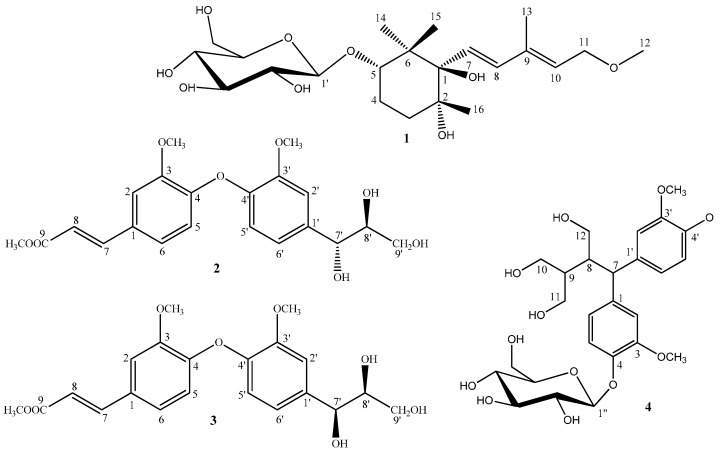
Structures of compounds **1**–**4**.

*Rehmannia glutinosa*, which belongs to Scrophulariaceae family, has been used as traditional Chinese herbal medicine for thousands of years. It was recorded in the Chinese medical classic “Shennong’s Herba” and was thought of in China as a “top grade” herb. Previous phytochemical studies on the dried and steamed roots of *R. glutinosa* have led to the isolation and identification of iridoid glycosides, ionone glycosides, phenethyl alcohol glycosides, and several other components [1,2,3,4,5]. In our previous studies, three new ursane-type triterpenes glutinosalactone **A**–**C** were isolated from the leaves, a new megastigmane, rehmamegastigmane, from the fresh roots, and two new ionone glycosides frehmaglutoside **G** and **H** from the dried roots [6,7,8]. Next, the further phytochemical study was undertaken to investigate the chemical constituents of the 95% EtOH extract of the dried roots of *R. glutinosa*, which led to the isolation of four new compounds: frehmaglutoside I (**1**) and rhemaneolignan **A**–**C** (**2**–**4**) (Figure 1). Their structures were elucidated by extensive analysis of HR-ESI-MS, 1D and 2D NMR, CD data, and chemical methods. In this paper, we describe the isolation, structure elucidation, and biological evaluation of the new compounds.

## 2. Results and Discussion

**Figure 2:**
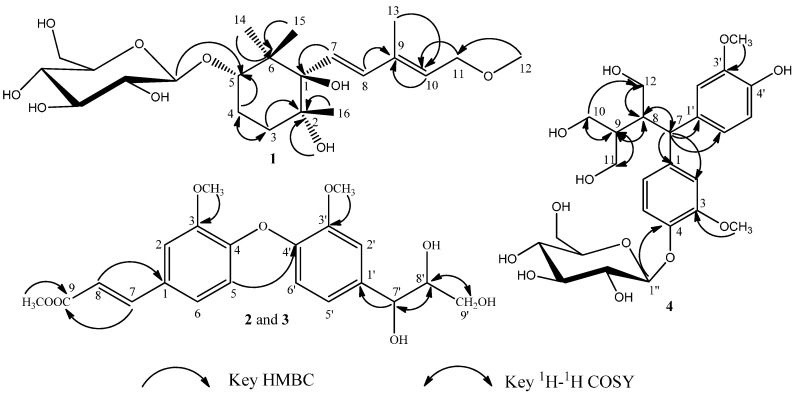
Key HMBC and ^1^H-^1^H COSY correlations of **1**–**4**.

Compound **1** was obtained as pale yellow crystalline powder, with a molecular formula of C_22_H_38_O_9_ on the basis of a [M + Na]^+^ ion peak at *m*/*z* 469.2407 (calcd. 469.2414) in the HRESIMS. It showed IR absorptions for hydroxyl (3361 cm^−1^), methyl (2923 cm^−1^), double bond (1698 cm^−1^) and ether linkages (1074 and 1025 cm^−1^). In the ^1^H- and ^13^C-NMR spectra of **1** (Table 1), the signal patterns were similar to those of frehmaglutoside H [8] from *R. glutinosa*, except for the presence of one methoxy (δ_H_ 3.32 (3H, s, OCH_3_) and δ_C_ 58.0 (OCH_3_)). The NMR chemical shifts at the C-11 position of **1** were shifted downfield to δ_H_ 4.15 (2H, d, *J* = 7.0 Hz, H-11) and δ_C_ 69.1 (C-11) compared with those of frehmaglutoside **H**, suggesting that the methoxy was located at the hydroxymethyl group of C-11. This finding was supported by the heteronuclear multiple-bond connectivity (HMBC) correlation from H-12 (δ 3.32) to C-11 (Figure 2).

**Table 1:** ^1^H- and ^13^C-NMR data for **1** (500 and 125 MHz, δ ppm).

No.	δ_H_	δ_C_	No.	δ_H_	δ_C_
1	-	82.3	12	3.32 (3H, s)	58.0
2	-	75.3	13	1.92 (3H, s)	21.0
3	1.96 (1H, m) 1.49 (1H, m)	35.9	14	1.17 (3H, s)	18.8
4	1.96 (2H, m) 1.88 (2H, m)	26.9	15	0.95 (3H, s)	22.6
5	3.73 (1H, m)	85.5	16	1.03 (3H, s)	27.0
6	-	45.0	1′	4.31 (1H, d, 7.5)	106.7
7	6.29 (1H, d, 16.0)	134.1	2′	3.16–3.64 (4H, m)	75.6
8	6.68 (1H, d, 16.0)	127.5	3′		78.2
9	-	137.5	4′		71.6
10	5.45 (1H, t, 6.5)	125.4	5′		77.7
11	4.15 (2H, d, 7.0)	69.1	6′	3.84 (1H, dd, 2.5, 11.0) 3.67 (1H, dd, 5.0, 11.0)	62.8

**Figure 3:**
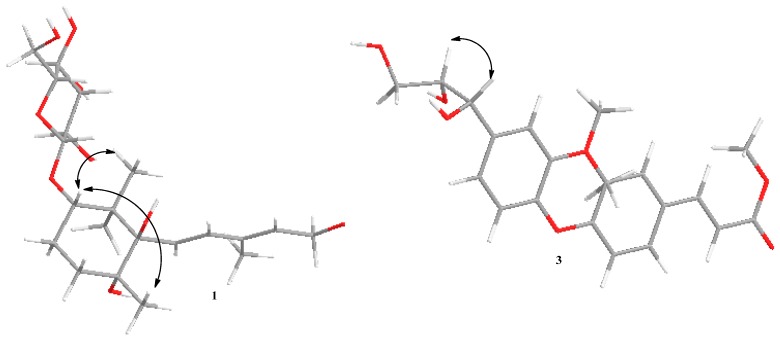
Key NOESY correlations of compounds **1** and **3**.

The relative configuration of **1** was confirmed by analysis of the NOESY spectrum, the correlations between H-5 (δ 3.73) and H-15 (δ 0.95)/H-16 (δ 1.03) revealed that they were β-oriented; on the other hand, the correlations between H-5 and H-14 (δ 1.17) indicated that these protons were α-oriented respectively (Figure 3).

The CD spectrum of **1** displayed positive Cotton effect at 204 nm and negative Cotton effect at 232 nm, which were also similar to frehmaglutoside H. Therefore the asymmetric centers of **1** had a 1*R*, 2*R*, 5*S* configuration. Finally, in the acid hydrolysis of **1**, d-glucose was obtained as confirmed by TLC comparison with a reference sample, and the configuration was determined by measurement of the optical rotation value. On the basis of the above analysis, **1** was identified as (7*E*,9*E*)-7-[(1*R*,2*R*,5*S*)-trihydroxy-2,6,6-trimethylcyclohexane]-9-methyipenta-7,9-dienoic-11-methoxy-5-*O*-β-d-glucopyranoside, and named frehmaglutoside I (Figure 1).

Compound **2** was isolated as a colorless amorphous powder with ${\left[\alpha \right]}_{D}^{20}$ −4.20 (*c* 0.20, MeOD). Its molecular formula, C_21_H_24_O_8_, was determined by the observation of a sodiated molecular ion peak at *m*/*z* 427.1367 (calcd. for C_21_H_24_O_8_Na, 427.1369) in the HRESIMS. The IR spectrum showed the presence of hydroxyl (3355 cm^−1^), methyl (2956, 2923 and 2853 cm^−1^), ester carbonyl (1635 cm^−1^), and methoxyl groups (1260 and 1022 cm^−1^). The ^1^H-NMR data of **2** (Table 2) indicated the presence of two ABX patterns (δ_H_ 6.95 (1H, d, *J* = 8.5 Hz, H-3), 7.06 (1H, dd, *J* = 2.0, 8.5 Hz, H-4), 7.15 (1H, d, *J* = 2.0 Hz, H-6) and 7.02 (1H, d, *J* = 2.0 Hz, H-3′), 6.83 (1H, dd, *J* = 2.0, 8.0 Hz, H-5′), 6.70 (1H, d, *J* = 8.0 Hz, H-6′)), *trans*-olefinic proton signals (δ_H_ 7.59 (1H, d, *J* = 16.0 Hz, H-7), 6.38 (1H, d, *J* = 16.0 Hz, H-8)), and three methoxy groups (δ_H_ 3.81 (3H, s), 3.77 (3H, s), 3.76 (3H, s)) [9]. The ^13^C-NMR and DEPT spectra (Table 2) of **2** showed 21 carbon signals, including three methoxyls (δ_C_ 52.0, 56.3, 56.6), one methylene (δ_C_ 62.4), ten methines (δ_C_ 74.1, 85.5, 112.0, 112.4, 115.6, 116.5, 117.6, 121.2, 123.5, 129.5), six quaternary carbons (δ_C_ 134.0, 146.3, 147.1, 148.7, 151.8, 151.8) and one ester carbonyl (δ_C_ 169.5). These ^1^H- and ^13^C-NMR data implied that compound **2** should be an oxyneolignane and were similar to those of 2,2′-dimethoxy-4-(3-hydroxypropenyl)-4′-(1,2,3-trihydroxypropyI) biphenyl ether [10], except for the fact the hydroxymethyl group (C-9) in it was replaced by an ester carbonyl (δ_C_ 169.5), and one additional methoxy unit was present in compound **2**. The HMBC experiment confirmed the abovementioned suggestion and placed the additional methoxyl unit at C-9 (Figure 2). The relative configuration of **2** was determined by analysis of the coupling constants. The H-7′ possessed a β-orientation on the basis of the observed *J* value of 6.0 Hz between H-7′ and H-8′, indicating that H-7′ and H-8′ were in a *trans*-configuration [11]. The NOESY correlations from H-8′ to H-7′ further supported the conclusion (Figure 3). Thus the relative configuration for **2** was determined. In addition, the absolute configuration of C-8′ was determined to be *S*, as the CD spectrum showed a positive Cotton effect at 253 nm [12,13]. Correspondingly, the absolute configuration of 7′ was elucidated as *R*. In summary, the structure of **2** was established as (7′*R*,8′*S*)- 3,3′-dimethoxy-1-(3-methl-1-acrylate)-1′-(1,2,3-trihydroxypropyl)-4-*O*-4′ neolignan and named rhemaneolignan A (Figure 1).

**Table 2:** ^1^H- and ^13^C-NMR data for **2** and **3** (500 and 125 MHz, δ ppm).

No.	2 (CD_3_OD)		3 (DMSO-*d*_6_)	
	δ_H_	δ_C_	δ_H_	δ_C_
1		151.8	-	149.5
2		151.8		150.8
3	7.15 (d, 2.0)	112.4	7.32 (d, 2.0)	111.2
4		129.5	-	126.7
5	7.06 (dd, 2.0, 8.5)	123.5	7.18 (dd, 2.0, 8.0)	122.6
6	6.95 (d, 8.5)	117.6	7.04 (d, 8.0)	114.4
7	7.59 (d, 16.0)	146.3	7.51 (d, 16.0)	144.7
8	6.38 (d, 16.0)	116.5	6.52 (d, 16.0)	115.1
9		169.5	-	167.0
OCH_3_	3.81 (s)	56.6	3.80 (s)	55.7
OCH_3_′′	3.76 (s)	52.0	3.69 (s)	51.2
1′	-	147.1	-	145.5
2′		148.7		147.0
3′	7.02 (d, 2.0)	112.0	6.95 (d, 2.0)	111.0
4′		134.0	-	132.9
5′	6.83 (dd, 2.0, 8.0)	121.2	6.74 (dd, 2.0, 8.0)	119.0
6′	6.70 (d, 8.0)	115.6	6.66 (d, 8.0)	114.6
7′	4.81 (d, 6.0)	74.1	4.68 (d, 4.0)	76.0
8′	4.48 (m)	85.5	4.37 (m)	83.8
9′	3.83 (m)	62.4	3.56 (m)	60.1
	3.76 (m)		3.23 (m)	
OCH_3_′	3.77 (s)	56.3	3.71 (s)	55.4

Compound **3**, isolated as a colorless powder with ${\left[\alpha \right]}_{D}^{20}$ +1.79 (*c* 0.20, MeOD), gave the same molecular formula C_21_H_24_O_8_ as that of **2** by positive HRESIMS (*m*/*z* 427.1366 [M + Na]^+^, calcd. for 427.1369) and was found to be an diastereoisomer of compound **2**. The two structures differ from each other only by the stereochemistry at the two chiral centers (C-7′ and C-8′). The ^1^H-NMR spectrum was almost identical to that of **2** except for the coupling constant of H-7′ (δ_H_ 4.68 (d, *J* = 4.0)), which suggested that H-7′ possessed a α-orientation, furthermore indicating that H-7′ and H-8′ were in a *cis*-configuration. The CD spectrum of 3 exhibited similar Cotton effects (positive at 258 nm) to that of **2**. Therefore the asymmetric centers of **3** were 7′*S*, 8′*S* configuration. Accordingly, the structure of **3** was defined as (7′*S*,8′*S*)-3,3′-dimethoxy-1-(3-methl-1-acrylate)-1′-(1,2,3-trihydroxypropyl)-4-*O*-4′-neo-lignan and named rhemaneolignan B (Figure 1).

Compound **4**, as pale yellow amorphous powder, was assigned the molecular formula C_26_H_36_O_12_ by HRESIMS (*m*/*z* 563.2101 [M + Na]^+^, calcd. for 563.2107). The NMR spectroscopic data of **4** (Table 3) was similar to that of daphneresinol [14], except for additional signals associated with a glucopyranosyl unit inferred from signals at δ 102.9 (C-1′′), 71.4 (C-2′′), 78.1 (C-3′′), 74.9 (C-4′′), 77.8 (C-5′′), 62.5 (C-6′′). In the ^1^H-NMR spectrum, the anomeric proton signal at δ 4.80 (1H, d, *J* = 8.0 Hz) pointed out the existence of one β-glucopyranose unit. In the HMBC spectrum, a correlation from the anomeric proton to C-4 (δ 146.1) indicated unequivocally that the β-glycopyranosyl moiety was located at C-4 (Figure 2). Consequently, the structure of **4** was identified as 4-*O*-β-d-glucopyranoside-2-benzhydryl-3-hydroxymethyl butane-1,4-diol and named rhemaneolignan C (Figure 1).

**Table 3:** ^1^H- and ^13^C-NMR data for **4** (500 and 125 MHz, δ ppm).

No.	δ_H_	δ_C_	No.	δ_H_	δ_C_
1	-	141.3	1′	-	136.7
2	7.02 (d, 2.0)	113.8	2′	6.97 (d, 2.0)	113.0
3	-	150.7	3′	-	149.1
4	-	146.1	4′	-	145.8
5	7.08 (d, 2.0)	118.2	5′	6.73 (d, 2.0)	116.3
6	6.95 (dd, 2.0, 8.0)	121.4	6′	6.86 (dd, 2.0, 8.0)	121.8
7	4.07 (d, 12.5)	52.1	gluʺ		
8	3.69 (2H, m)	44.7	1ʺ	4.80 (d, 8.0)	102.9
9	1.96 (2H, m)	43.7	2ʺ	3.38 (1H, m)	71.4
10	3.75 (2H, m)	63.6	3ʺ	3.38 (1H, m)	78.1
11	3.57 (1H, m)	60.0	4ʺ	3.47 (1H, m)	74.9
	3.38 (1H, m)				
12	3.75 (1H, m)	59.7	5ʺ	3.48 (1H, m)	71.8
	3.39 (1H, m)				
OCH_3_	3.87 (3H, s)	56.8	6ʺ	3.86 (1H, 2.0, 12.0)	62.5
				3.70 (1H, 6.0, 11.0)	
OCH_3_′	3.86 (3H, s)	56.5			

**Figure 4:**
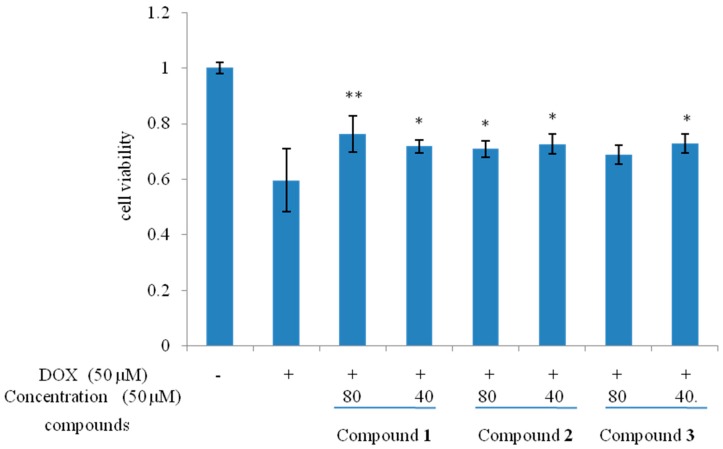
Protective effects of **1**–**3** on DOX-induced cytotoxicity in H9c2 cell. *n* = 3; * *p* < *0.*05; ** *p <* 0.01 compared with the DOX group.

To investigate whether compounds **1**–**3** could protect cells from DOX-induced cell death, H9c2 cells were treated with 50 μM DOX in the presence or absence of compounds **1**–**3** (40 and 80 μM) and the cell viability was assessed by performing MTT assay. Among them, compound **1**–**3** exhibited protective effects against DOX-induced cardiotoxicity with different concentrations (Figure 4).

## 3. Experimental Section

### 3.1. General Experimental Procedures

NMR spectra (500 MHz for ^1^H-NMR and 125 MHz for ^13^C-NMR) were recorded in CD_3_OD or DMSO-*d*_6_ on an AVANCE III 500 spectrometer (Bruker Daltonics, Bremen, Germany), with TMS as internal standard. Optical rotations were measured with an AP-IV instrument (Rudolph Research Analytical, Hackettstown, Madison, WI, USA). IR spectra were determined on a Nicolet iS 10 Microscope Spectrometer (Thermo Scientific, San Jose, CA, USA). HR-ESI-MS spectra were recorded on a Bruker maxis HD mass spectrometer. UV spectra were recorded on a UV-2401PC apparatus (Shimadzu Corporation, Kyoto, Japan). HPLC was performed on a Waters Alliance 2695 separations module (equipped with Empower software) connected to a Waters 2998 photodiode array (PDA) detector (190–800 nm) equipped with a Platisil ODS C18 column (250 mm × 4.6 mm I.D. 5 μm) (Waters, Milford, MA, USA). Preparative HPLC was conducted using a Saipuruisi LC-50 instrument with an UV200 detector (Beijing, China) and a YMC-Pack ODS-A column (250 mm × 20 mm, 5 μm and 250 mm × 10 mm, 5 μm). Column chromatography was made use of Diaion HP-20 (Mitsubishi Chemical Corporation, Tokyo, Japan), Toyopearl HW-40, MCI gel CHP-20 (TOSOH Corp., Tokyo, Japan), Sephadex LH-20 (40–70 μm, Amersham Pharmacia Biotech AB, Uppsala, Sweden), silica gel (160–200 mesh, Marine Chemical Industry, Qingdao, China). TLC was carried out on self-made silica gel G (Marine Chemical Industry) plates, CH_2_Cl_2_/MeOH/H_2_O (10:1:0.1, *v*/*v*), CH_2_Cl_2_/MeOH/H_2_O (4:1:0.1, *v*/*v*) as the eluent, and spots were visualized by spraying with 10% H_2_SO_4_ in ethanol (*v*/*v*) followed by heating. The chemical reagents were supplied by Beijing Chemical Plant (Beijing, China), Tianjin NO. 3 Reagent Plant (Tianjin, China) and Tianjin Shield Fine Chemicals Company (Tianjin, China).

### 3.2. Plant Material

The dried roots of *R. glutinosa* were collected from Jiaozuo, Henan Province in China, in November 2010. The plants were identified by Prof. Chengming Dong of Henan University of TCM. A voucher specimen (No. 20101101A) has been stored in the Department of Natural Medicinal Chemistry, School of Pharmacy, Henan University of TCM, Zhengzhou, China.

### 3.3. Extraction and Isolation

The dried roots (20.0 kg) of *R.* *glutinosa* were cut into small pieces and extracted twice with 95% EtOH (200 L) under reflux for 2 h. After concentrating under reduced pressure on a vacuum evaporator, the concentrated solution (1500 mL) was centrifuged and subjected to Diaion HP-20 column chromatography, eluting successively with H_2_O and MeOH, to afford water and methanol extracts (101.6 g). Next the methanol extracts were dissolved in 10% MeOH/H_2_O (1000 mL), and subjected to Diaion HP-20 column chromatography once again, eluting successively with 10%, 30%, 50%, 70%, 100% (*v*/*v*) MeOH/H_2_O (10 column volumes each). After removing the solvents, extracts of 65.0 g, 16.6 g, 11.5 g, 4.2 g, 2.0 g, were obtained, respectively.

The 10% MeOH/H_2_O (65.0 g) fraction was subjected to silica gel column chromatography eluting with CH_2_Cl_2_/MeOH (30:1, 20:1, 10:1, 5:1) to afford Fr. 1.1–1.4. Fr. 1.3 (8.6 g) was purified over Sephadex LH-20 using MeOH/H_2_O (4:6) to afford Fr. 2.1–2.4. Fr. 2.2 was separated by preparative HPLC with MeOH/H_2_O (33:67) (YMC-Pack ODS-A, Kyoto, Japan, 10 mm × 250 mm) to afford compound **1** (11 mg).

The 30% MeOH/H_2_O (16.6 g) fraction was subjected to Toyopearl HW-40 column chromatography eluting with MeOH/H_2_O from 0% to 100% to give Frs. 1.1–1.4. Fr. 1.4 was fractionated repeatedly by column chromatography on MCI gel CHP-20 using MeOH/H_2_O (2:8) to yield Frs. 2.1–2.5. Fr. 2.4 was repeatedly chromatographed on a silica gel column with CH_2_Cl_2_/MeOH (10:1) to yield **4** (3.5 mg).

The 50% MeOH/H_2_O (11.5 g) fraction was fractionated on a silica gel column and eluted with mixtures of CH_2_Cl_2_ and MeOH of increasing polarity to afford fractions 1.1–1.6. Fraction 3 was chromatographed on a Sephadex LH-20 column eluting with MeOH/H_2_O (7:3), followed by purification by semi-preparative HPLC with MeOH/H_2_O (52:48) at a flow rate of 3 mL/min to give compounds **2** (12.0 mg) and **3** (8.6 mg).

### 3.4. Spectral Data

*Frehmaglutoside I* (**1**): Pale yellow crystalline powder; ${\left[\alpha \right]}_{D}^{20}$ −13.7 (*c* 0.20, CH_3_OH); UV_max_ 237 nm; CD (MeOH): 204 (Δε +3.09), 232 (Δε −5.23) nm; IR (MeOH)ν_max_: 3361, 2923, 2854, 1698, 1613, 1453, 1074, 1025 cm^−1^; HRESIMS: *m*/*z* 469.2407 [M + Na]^+^, C_22_H_38_O_9_; ^1^H-NMR (500 MHz, CD_3_OD) spectral data and ^13^C-NMR (125 MHz, CD_3_OD) spectral data, see Table 1.

*Rhemaneolignan **A*** (**2**): Colorless amorphous powder, ${\left[\alpha \right]}_{D}^{20}$ −4.20 (*c* 0.20, MeOD); UV_max_ 325 nm; CD (MeOH): 253 (Δε +2.12) nm; IR (MeOH)ν_max_: 3356, 2956, 2923, 2853, 1635, 1508, 1456, 1259, 1022 cm^−1^; HRESIMS: *m*/*z* 427.1367 [M + Na]^+^, C_21_H_24_O_8_; ^1^H-NMR (500 MHz, CD_3_OD) spectral data and ^13^C-NMR (125 MHz, CD_3_OD) spectral data, see Table 2.

*Rhemaneolignan **B*** (**3**): Colorless amorphous powder, ${\left[\alpha \right]}_{D}^{20}$ +1.79 (*c* 0.20, MeOD); UV_max_ 322 nm; CD (MeOH): 258 (Δε +2.73) nm; IR (MeOH)ν_max_: 3363, 2956, 2923, 2853, 1635, 1600, 1508, 1457, 1259, 1032 cm^−1^; HR-ESI-MS: *m*/*z* 427.1366 [M + Na]^+^, C_21_H_24_O_8_; ^1^H-NMR (500 MHz, DMSO-*d*_6_) spectral data and ^13^C-NMR (125 MHz, DMSO-*d*_6_) spectral data, see Table 2.

*Rhemaneolignan **C*** (**4**): Pale yellow amorphous powder, ${\left[\alpha \right]}_{D}^{20}$ −35.6 (*c* 0.14, MeOD); UV (MeOH) λ_max_: 278.3 nm; IR (MeOH)ν_max_: 3397, 2924, 2851, 1601, 1513, 1384, 1268, 1073, 1029; HR-ESI-MS *m*/*z* 563.2021 [M + Na]^+^, C_26_H_36_O_12_; ^1^H-NMR (500 MHz, CD_3_OD) spectral data and ^13^C-NMR (125 MHz, CD_3_OD) spectral data, see Table 3.

### 3.5. Activity Assay

#### 3.5.1. Cell Culture

The rat cardiac H9c2 myocardial cells, were spontaneously immortalized ventricular rat embryo myoblasts, were purchased from Biowit Technologies (Shenzhen, China). The cells were maintained in Dulbecco’s modified Eagle’s medium (DMEM) supplemented with 10% fetal bovine serum at 37 °C in a water-saturated 5.0% CO_2_ incubator.

#### 3.5.2. Measurement of Cell Viability

Cells were split when a confluence of ~80% was achieved using trypsin-EDTA and seeded onto 96-well plates at a density of 1.0 × 10^4^ cells∙L^−1^ (200 μL/well), incubated for 24 h before treatment. Thereafter, the cells were exposed to DOX (50 μM) for 24 h and then incubated in a fresh medium with compound **1**–**3** (80 and 40 μM) for an additional 24 h. The effects of compounds **1**–**3** on DOX induced cytotoxicity were assessed using the MTT assay, as previously described [15,16]. The optical density of each well was then measured on a microplate spectrophotometer at a wavelength of 490 nm. Cell viability was determined as the percentage of surviving cells compared with that of the DOX-treated control.

#### 3.5.3. Statistical Analysis

Experiments were performed in triplicate, and the values are the averages of three (*n* = 4) independent experiments. Individual data were expressed as mean ± standard deviation (SD). A *post-hoc* Dunnett’s test was used to obtain corrected *p*-values in group comparisons. Statistical analyses were performed with one-way ANOVA (SPSS version 13.0). A *p* value of 0.05 or less was considered significant.

## 4. Conclusions

In conclusion, a new ionone glycoside. frehmaglutoside I (**1**). and three new rhemaneolignans **A**–**C** (**2**–**4**), were isolated from the dried roots of *Rehmannia glutinosa*. Among them, different concentrations of compounds **1**–**3** exhibited protective effects against DOX-induced cardiotoxicity.

## Supplementary Materials

The IR, HR-ESI-MS, NMR (1D and 2D) and CD spectra of compounds **1**–**3** are available as supporting information. Supplementary materials can be accessed at: http://www.mdpi.com/1420-3049/20/08/15192/s1.
