# Supplementary material for: A New Ionone Glycoside and Three New Rhemaneolignans from the Roots of Rehmannia glutinosa
Source: Molecules. 2015 Aug 20;20(8):15192–201. doi: 10.3390/molecules200815192 (PMC6332001; doi:10.3390/molecules200815192)
Supplement: Supplementary file 1 [file molecules-20-15192-s001.pdf]

Supplementary Materials

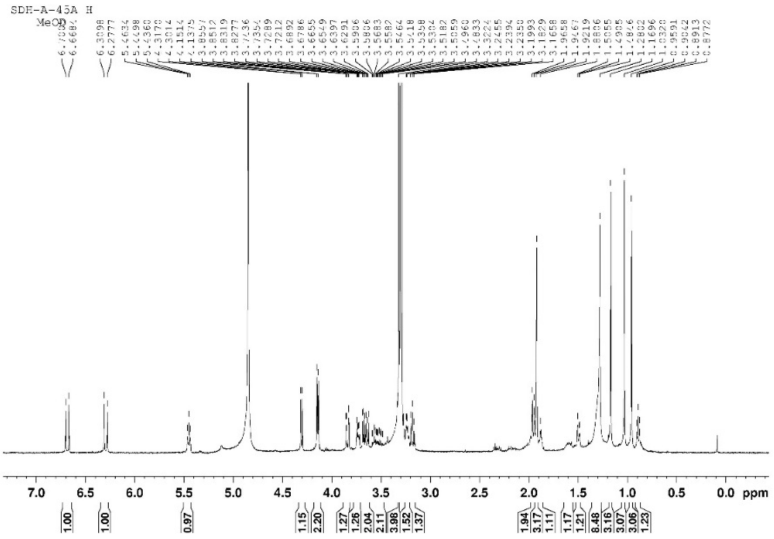

Figure S1. The ^{1}H-NMR spectrum of compound **1**.

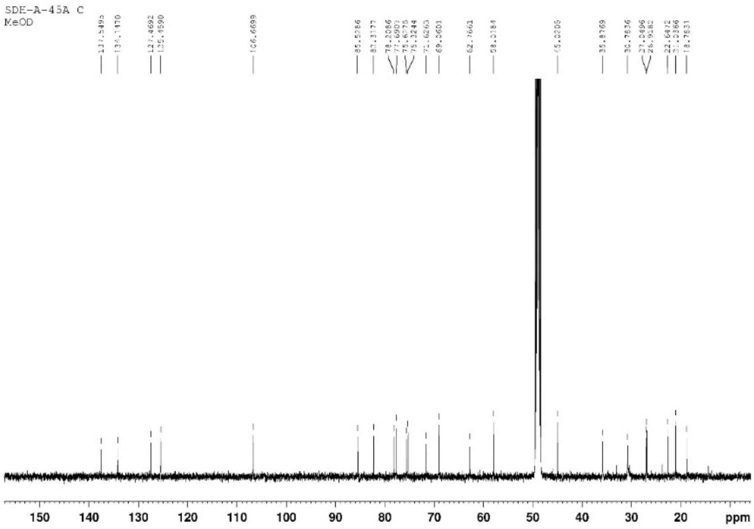

Figure S2. The ^{13}C-NMR spectrum of compound **1**.

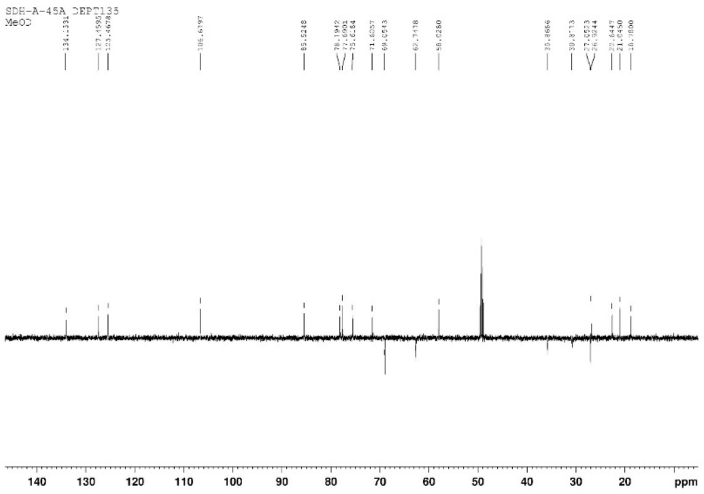

Figure S3. The DEPT135 spectrum of compound **1**.

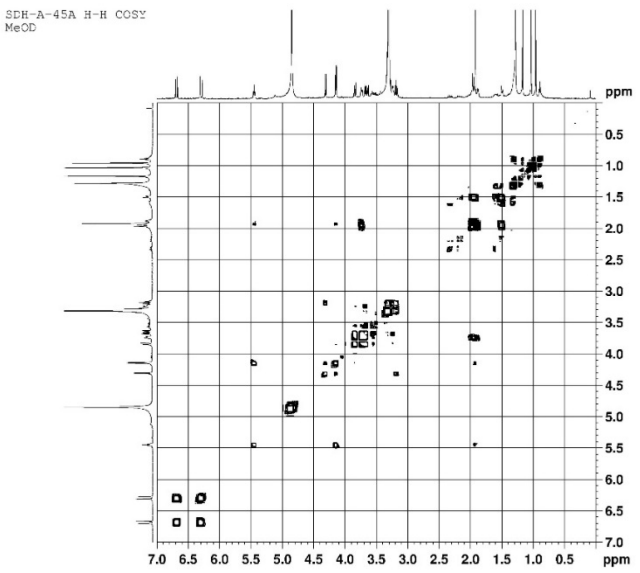

Figure S4. The ^{1}H-^{1}H COSY spectrum of compound **1**.

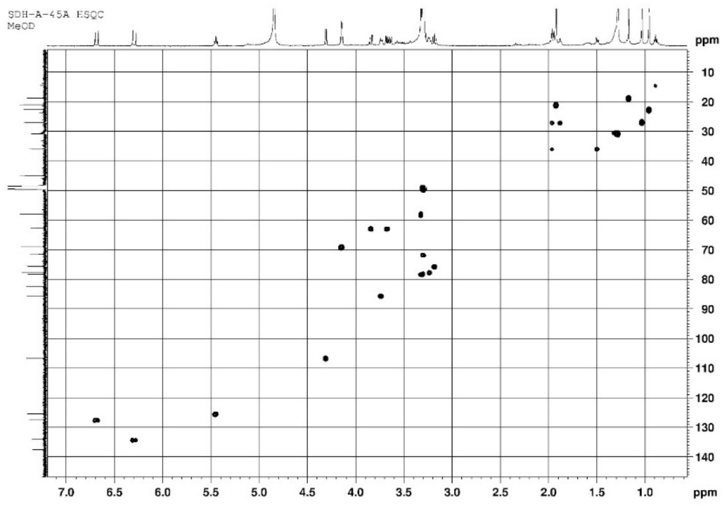

Figure S5. The HSQC spectrum of compound **1**.

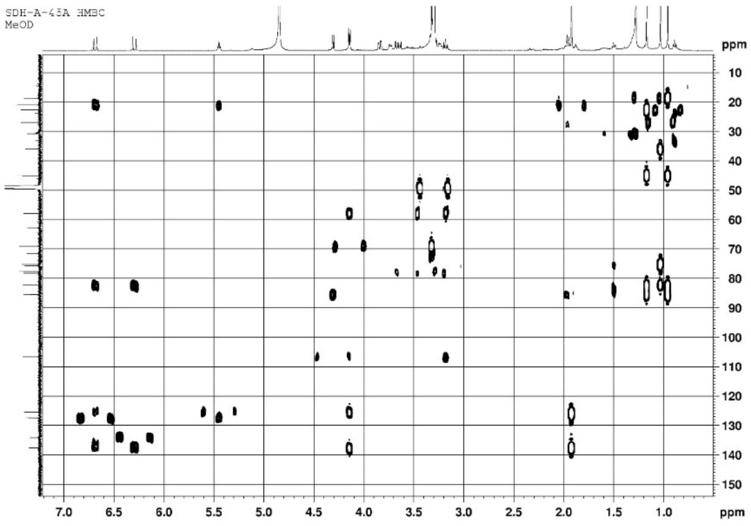

Figure S6. The HMBC spectrum of compound **1**.

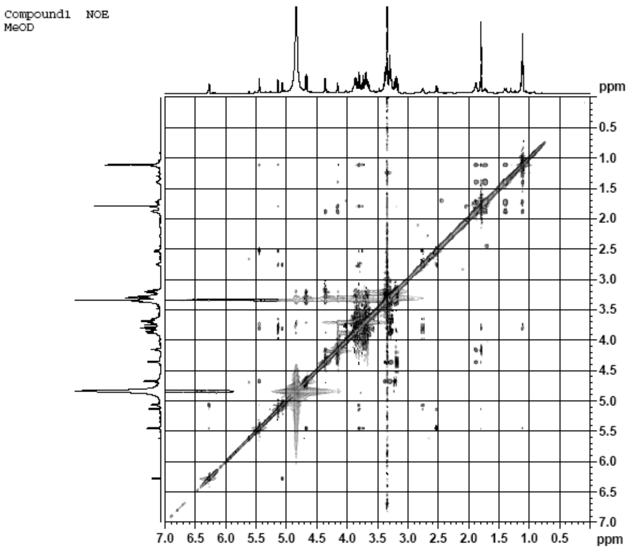

Figure S7. The NOESY spectrum of compound **1**.

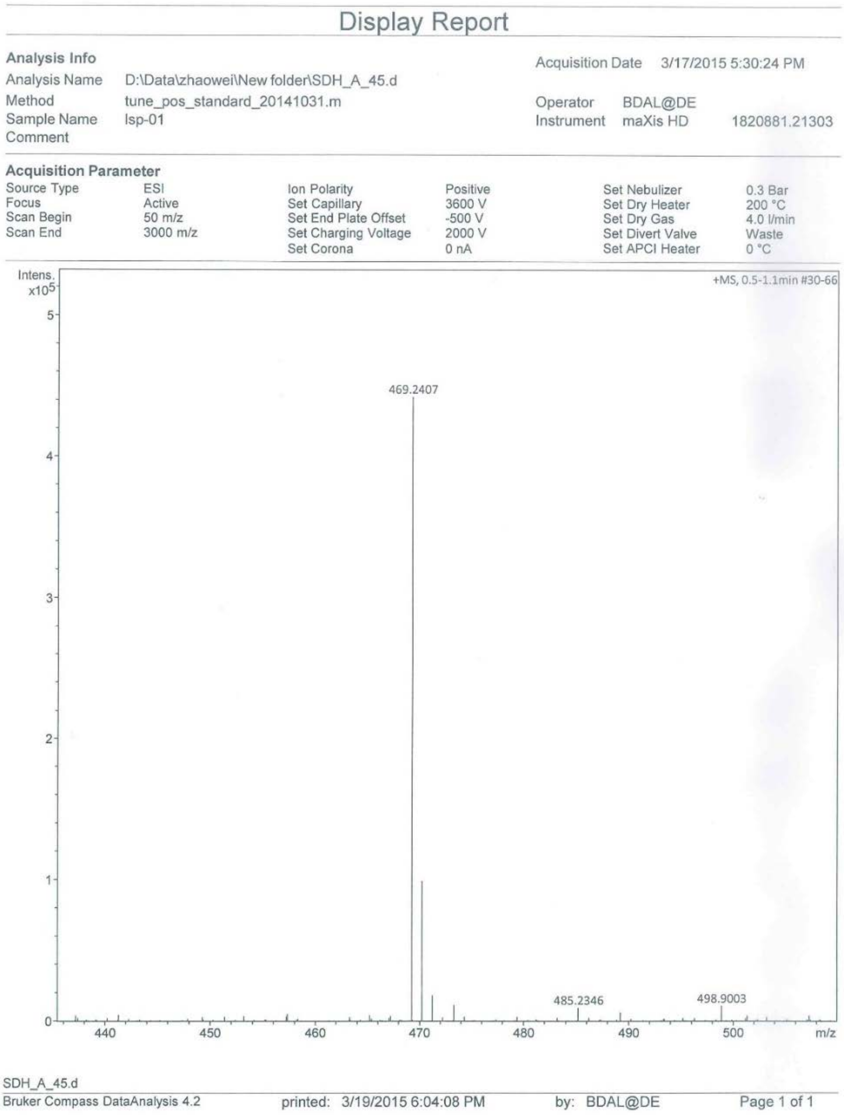

Figure S8. The HR-TOF-MS spectrum of compound **1**.

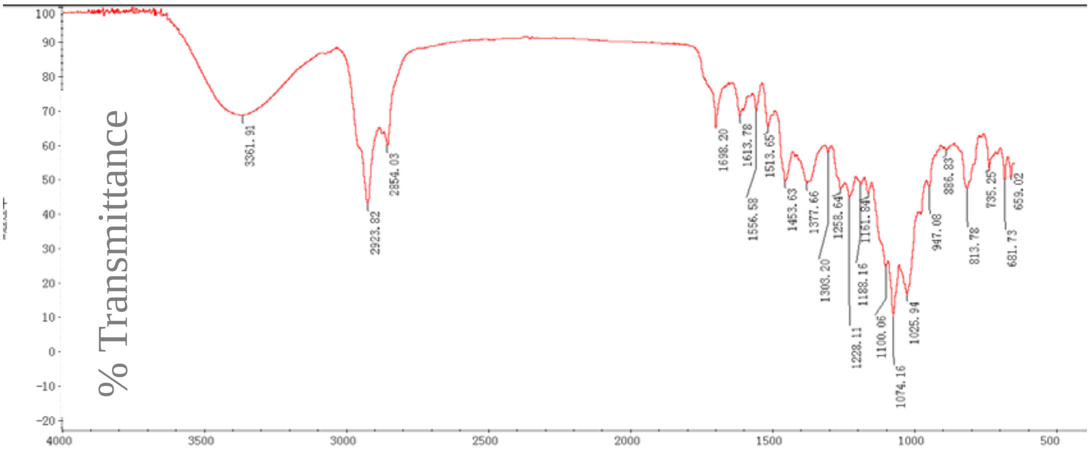

Figure S9. The IR spectrum of compound **1**.

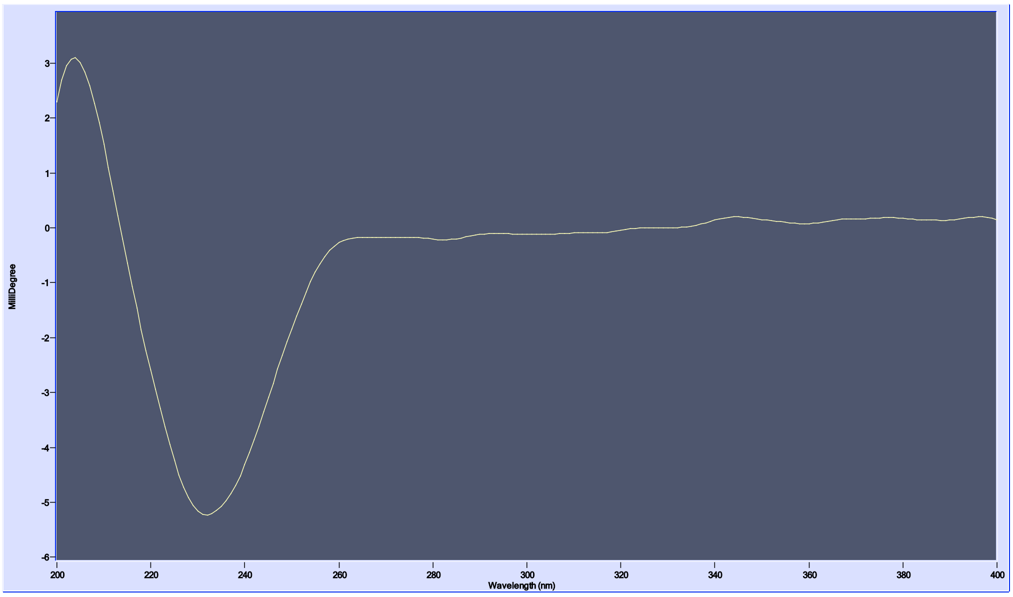

Figure S10. The CD spectrum of compound **1**.

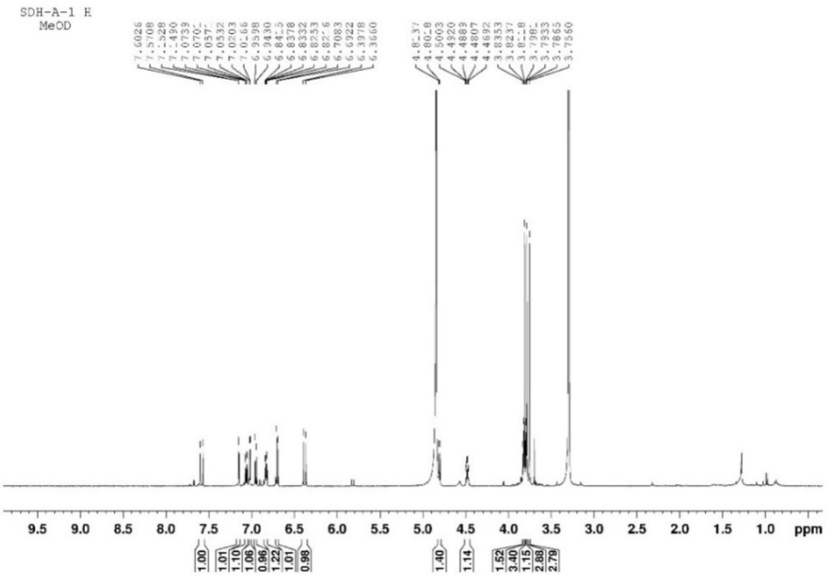

Figure S11. The ^{1}H-NMR spectrum of compound **2**.

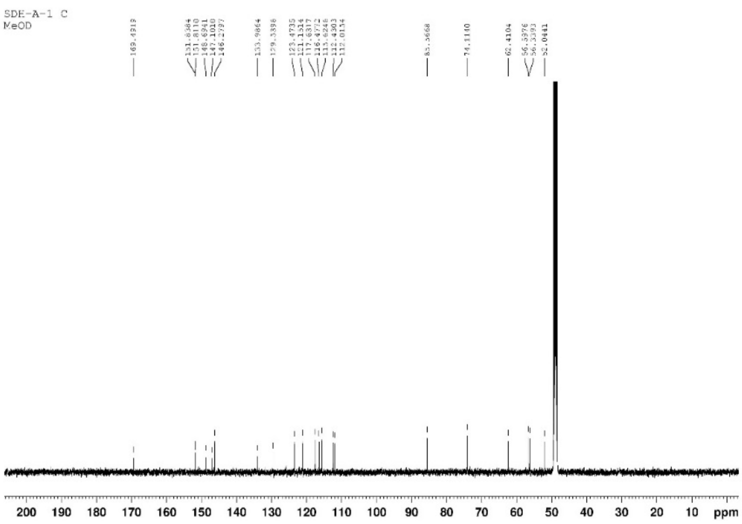

Figure S12. The ^{13}C-NMR spectrum of compound **2**.

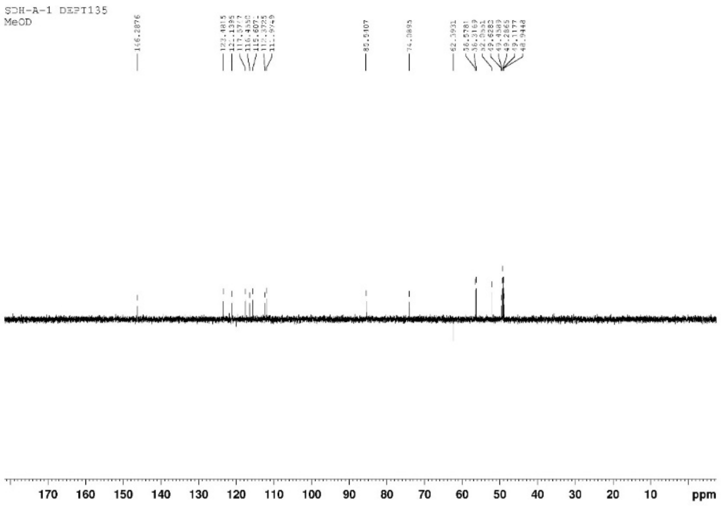

Figure S13. The DEPT135 spectrum of compound **2**.

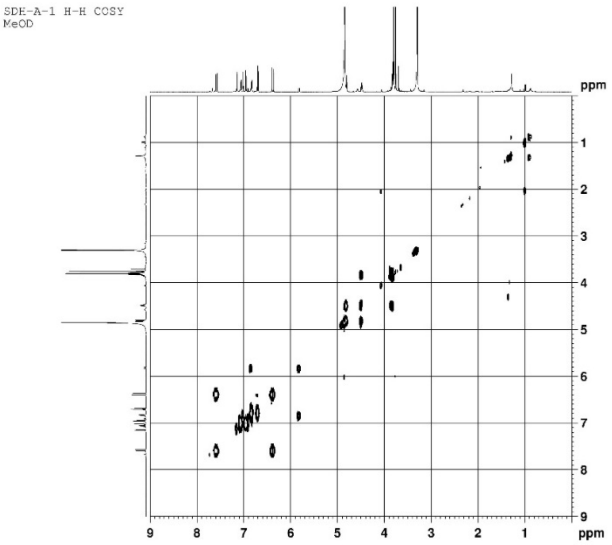

Figure S14. The ^{1}H-^{1}H COSY spectrum of compound **2**.

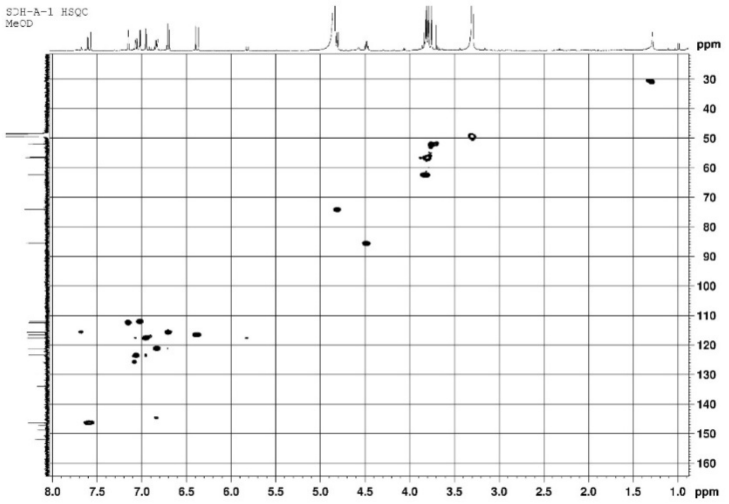

Figure S15. The HSQC spectrum of compound **2**.

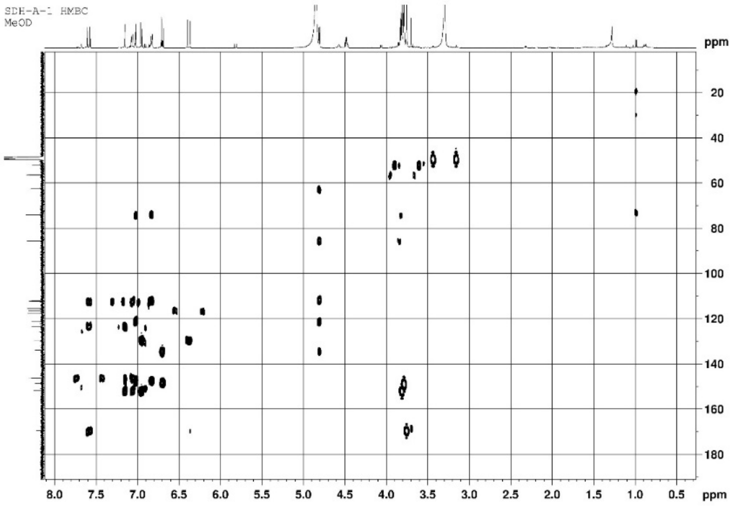

Figure S16. The HMBC spectrum of compound **2**.

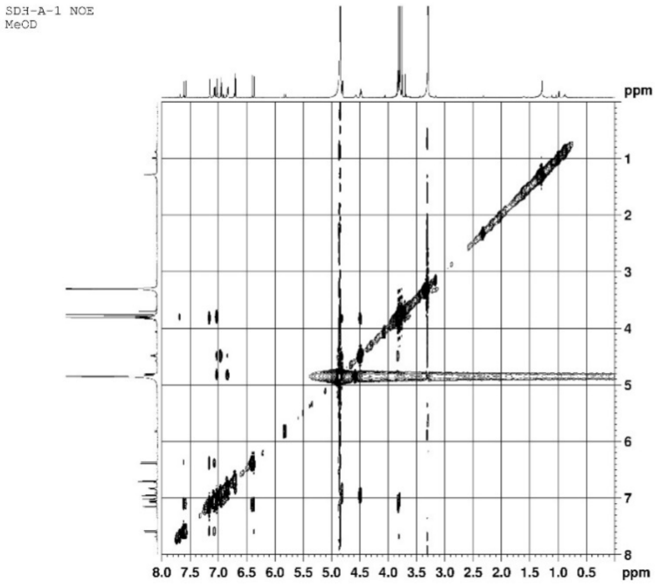

Figure S17. The NOESY spectrum of compound **2**.

Display Report

Analysis Info

Analysis Name	\\MQHD21303\Data\LM\DATA\SDH-A-1.d	Acquisition Date	2015/3/20 18:04:07
Method	tune_pos_standard_20141031.m	Operator	BDAL@DE
Sample Name	SDH-A-1	Instrument	maXis HD 1820881.21303
Comment			

Acquisition Parameter

Source Type	ESI	Ion Polarity	Positive	Set Nebulizer	0.3 Bar
Focus	Active	Set Capillary	3600 V	Set Dry Heater	200 ¡ãC
Scan Begin	50 m/z	Set End Plate Offset	-500 V	Set Dry Gas	4.0 l/min
Scan End	3000 m/z	Set Charging Voltage	2000 V	Set Divert Valve	Waste
		Set Corona	0 nA	Set APCI Heater	0 ¡ãC

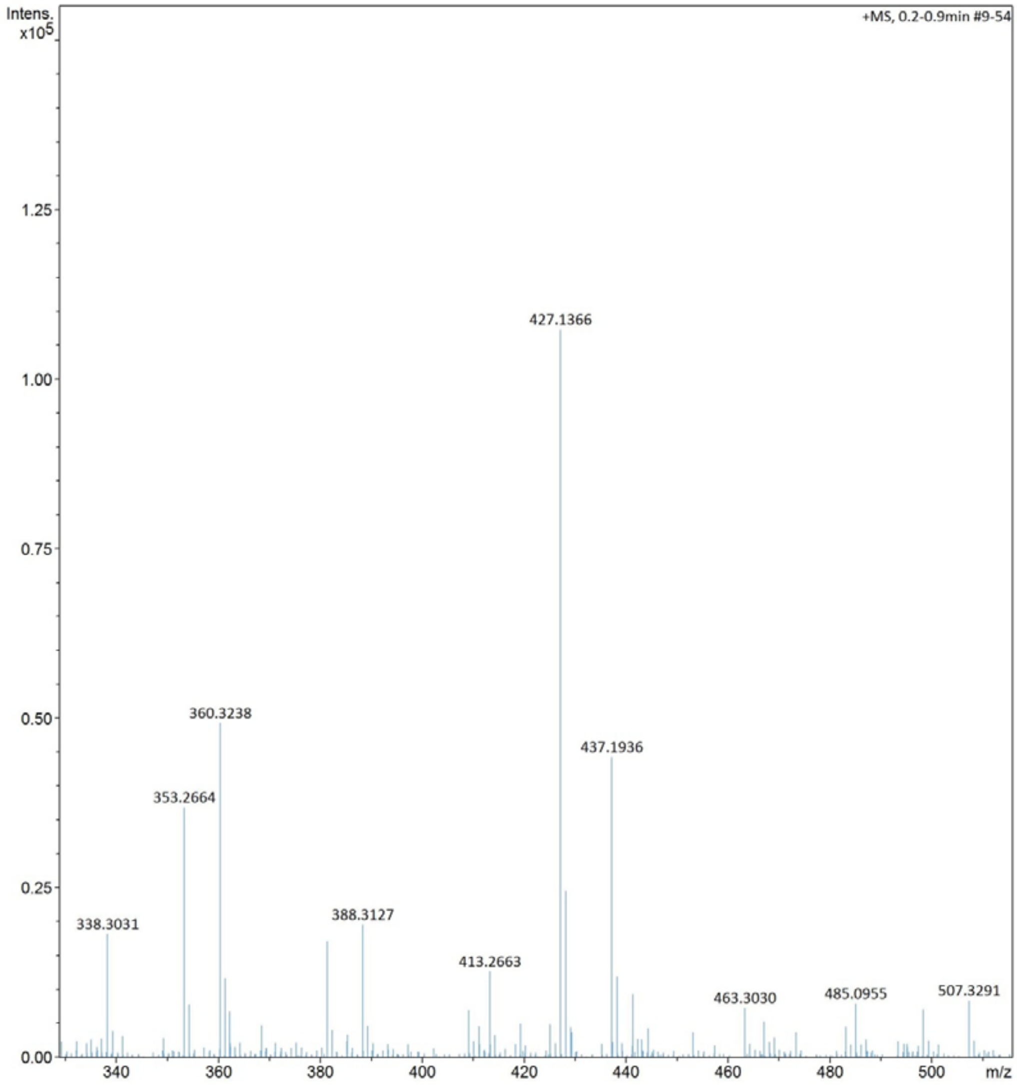

SDH-A-1.d

Bruker Compass DataAnalysis 4.2 printed: 2015/6/9 15:25:13 by: ZMH Page 1 of 1

Figure S18. The HRESIMS spectrum of compound **2**.

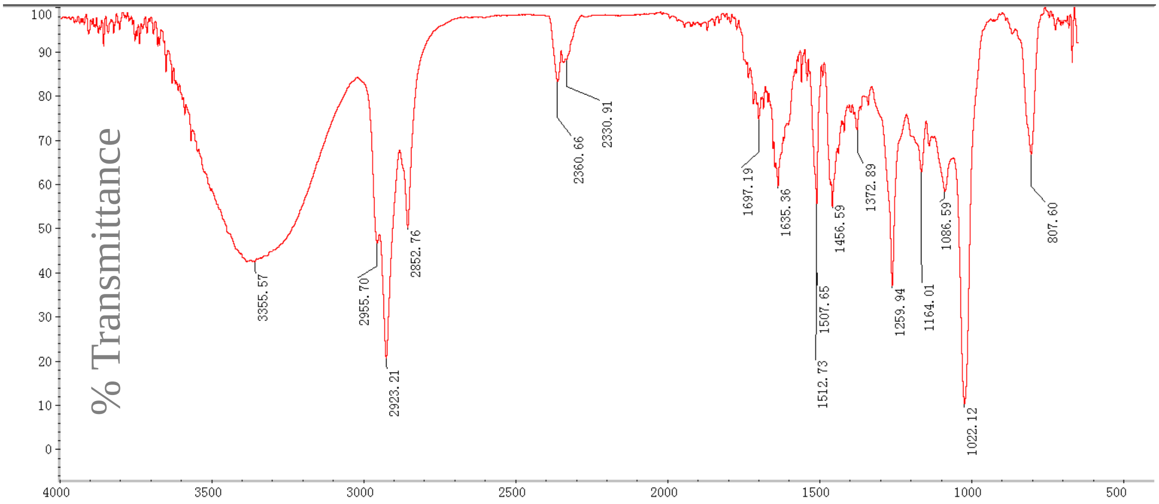

Figure S19. The IR spectrum of compound **2**.

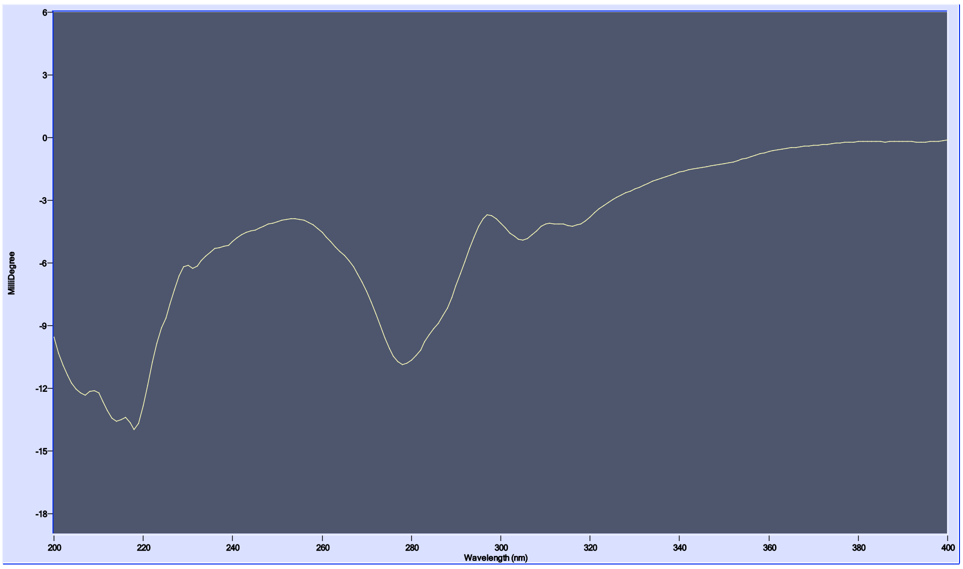

Figure S20. The CD spectrum of compound **2**.

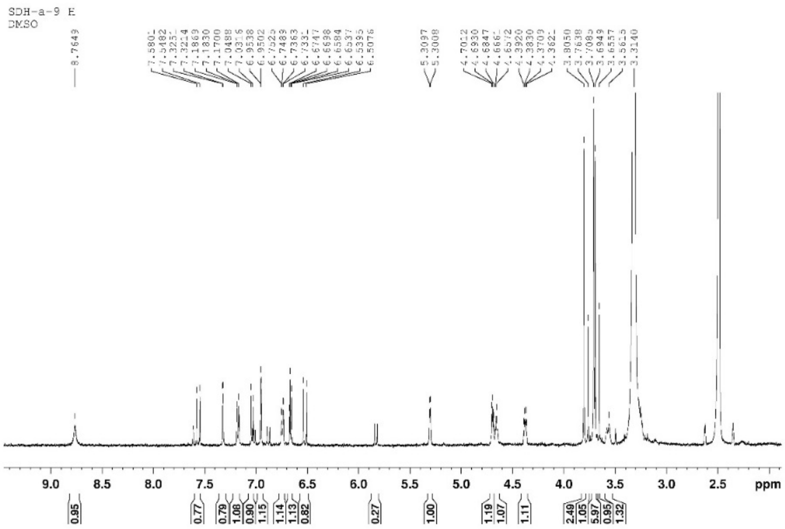

Figure S21. The ^{1}H-NMR spectrum of compound **3**.

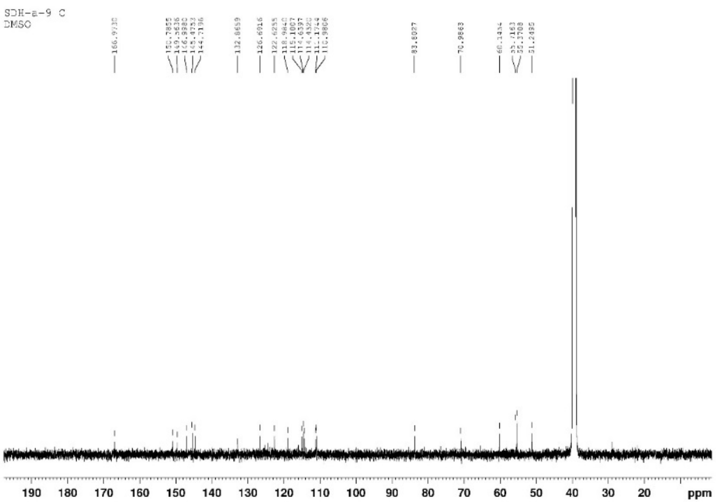

Figure S22. The ^{13}C-NMR spectrum of compound **3**.

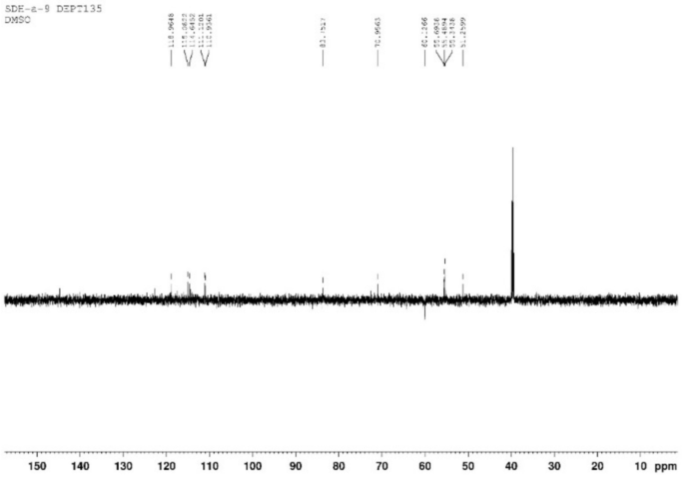

Figure S23. The DEPT135 spectrum of compound **3**.

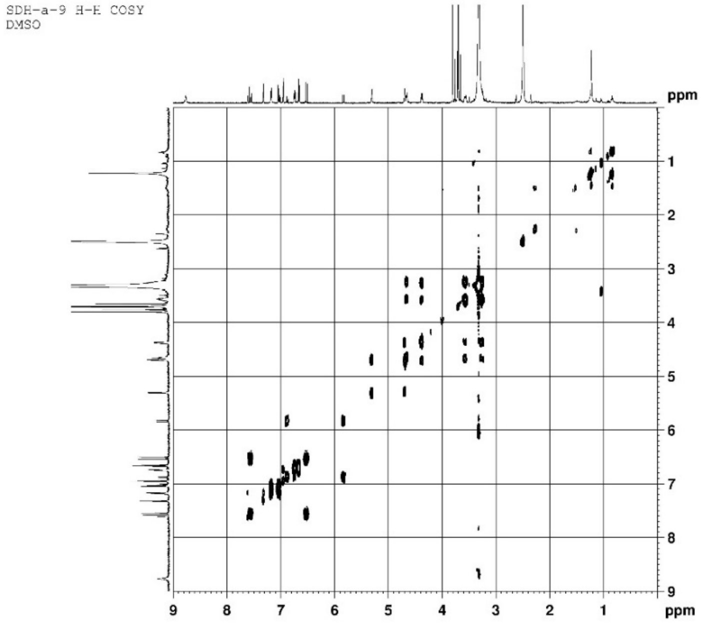

Figure S24. The ^{1}H-^{1}H COSY spectrum of compound **3**.

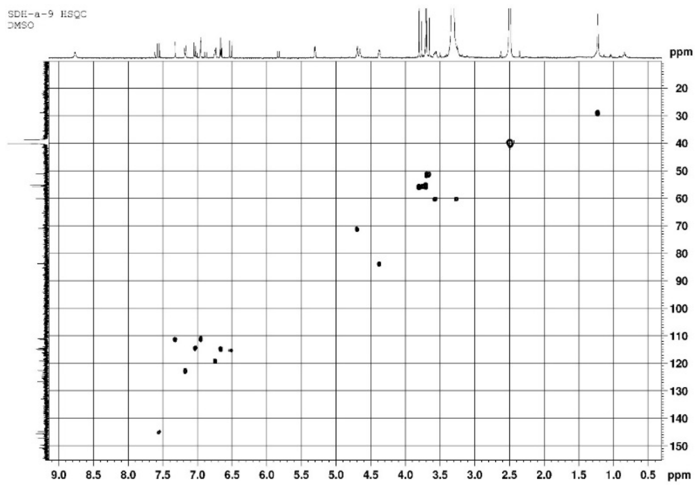

Figure S25. The HSQC spectrum of compound **3**.

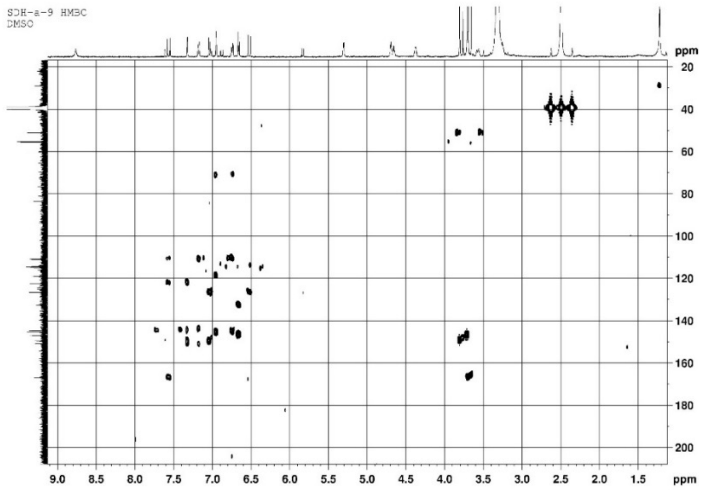

Figure S26. The HMBC spectrum of compound **3**.

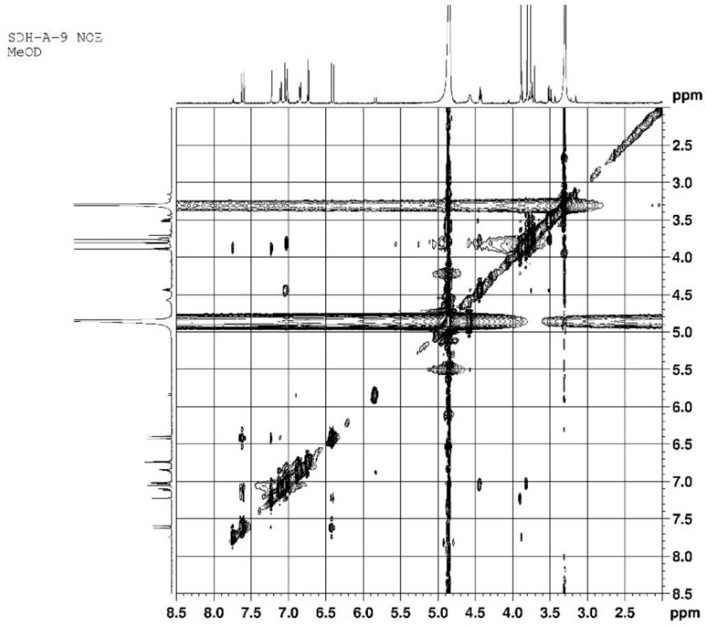

Figure S27. The NOESY spectrum of compound **3**.

Display Report

Analysis Info

Analysis Name	\\MQHD21303\Data\LM\DATA\SDH-A-9a.d	Acquisition Date	2015/3/20 18:03:05
Method	tune_pos_standard_20141031.m	Operator	BDAL@DE
Sample Name	SDH-A-9a	Instrument	maXis HD 1820881.21303
Comment			

Acquisition Parameter

Source Type	ESI	Ion Polarity	Positive	Set Nebulizer	0.3 Bar
Focus	Active	Set Capillary	3600 V	Set Dry Heater	200 ¡ãC
Scan Begin	50 m/z	Set End Plate Offset	-500 V	Set Dry Gas	4.0 l/min
Scan End	3000 m/z	Set Charging Voltage	2000 V	Set Divert Valve	Waste
		Set Corona	0 nA	Set APCI Heater	0 ¡ãC

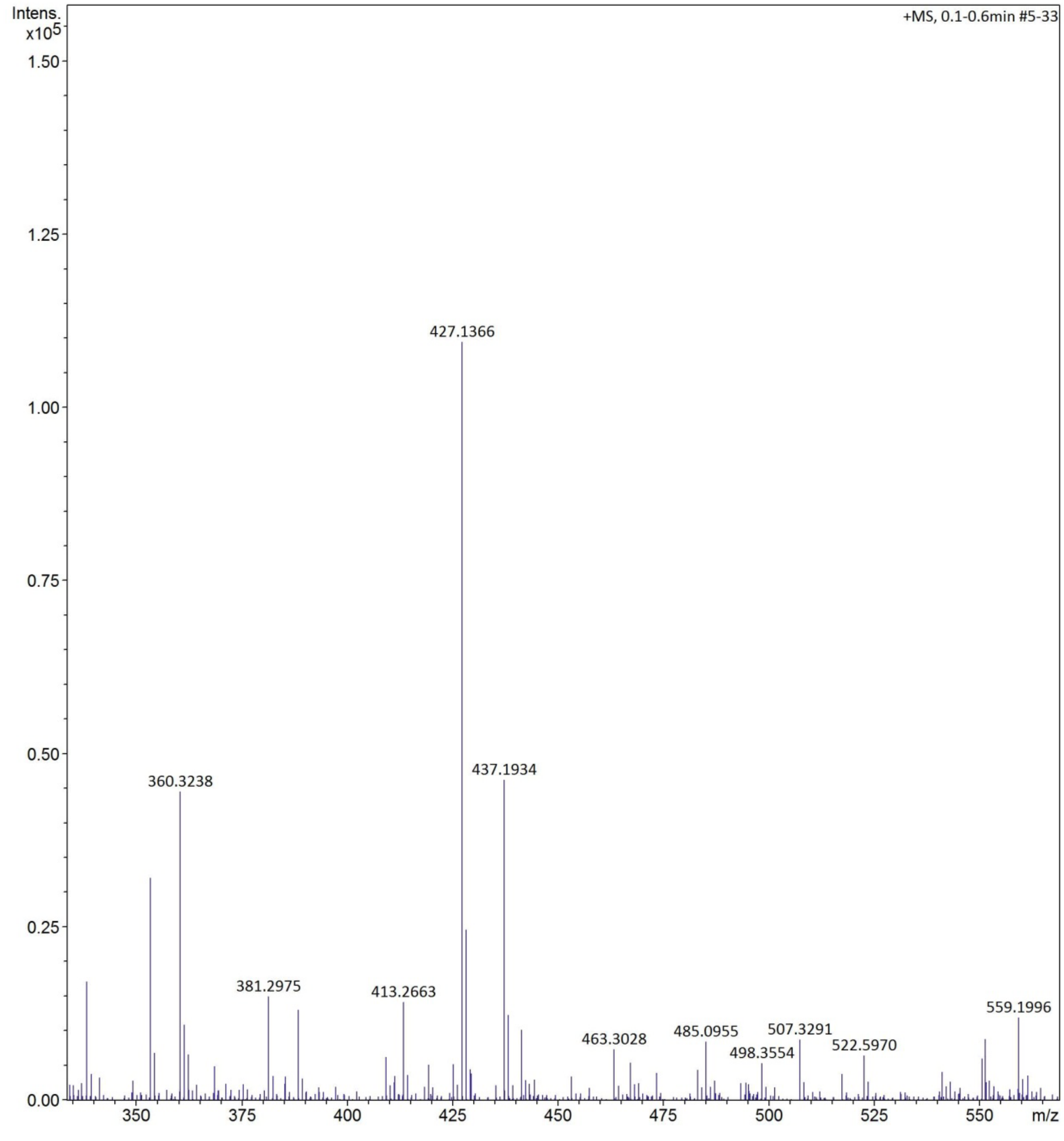

SDH-A-9a.d

Bruker Compass DataAnalysis 4.2 printed: 2015/6/10 19:09:35 by: ZMH Page 1 of 1

Figure S28. The HRESIMS spectrum of compound **3**.

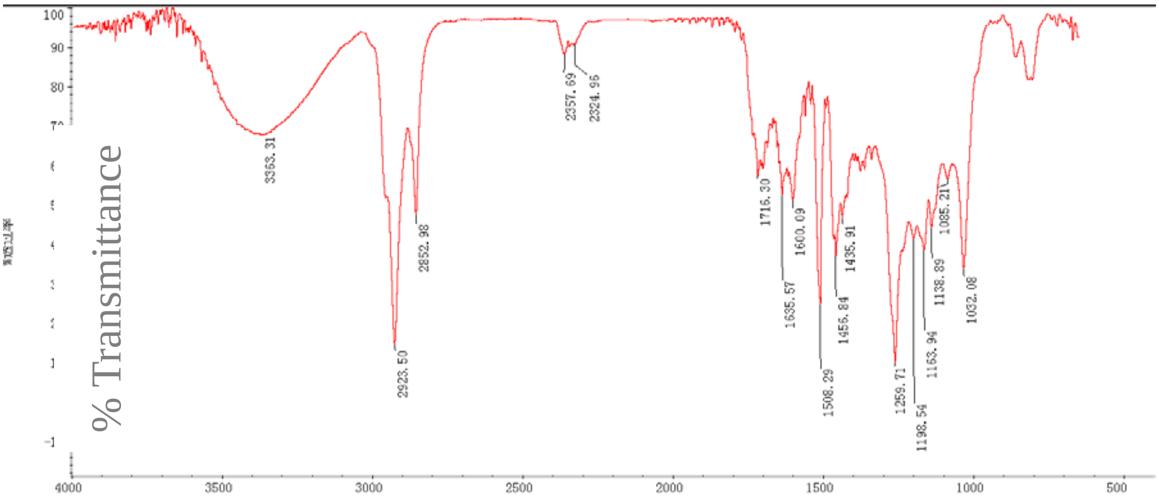

Figure S29. The IR spectrum of compound **3**.

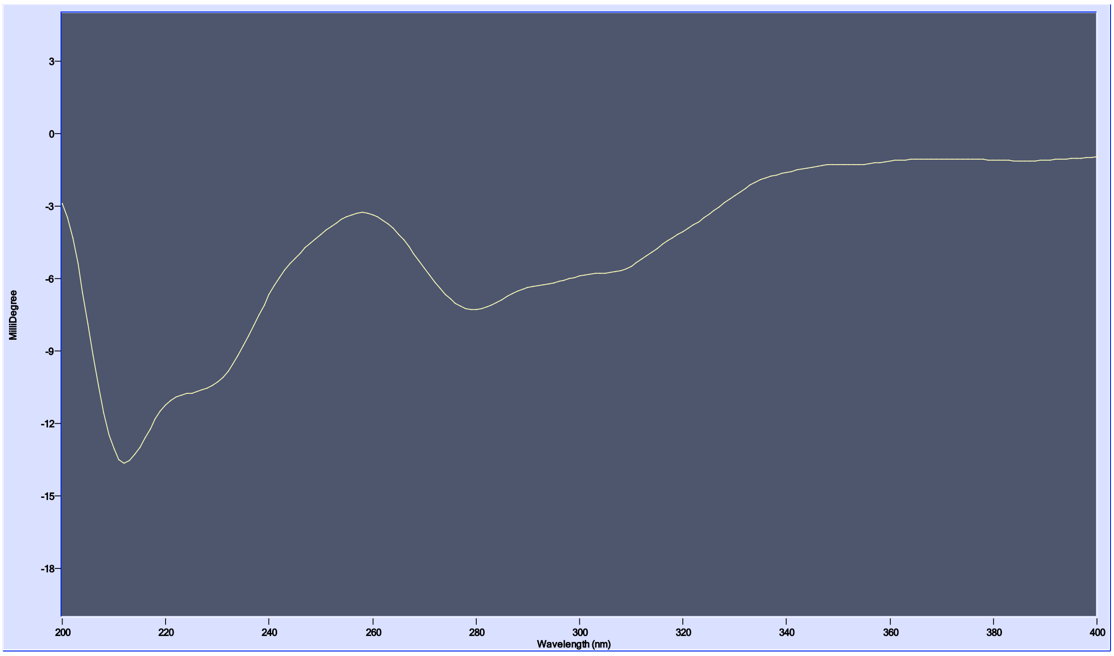

Figure S30. The CD spectrum of compound **3**.
